# The possible mediatory role of adipokines in the association between low carbohydrate diet and depressive symptoms among overweight and obese women

**DOI:** 10.1371/journal.pone.0257275

**Published:** 2021-09-13

**Authors:** Leila Setayesh, Reyhane Ebrahimi, Sara Pooyan, Habib Yarizadeh, Elaheh Rashidbeygi, Negin Badrooj, Hossein Imani, Khadijeh Mirzaei

**Affiliations:** 1 Department of Community Nutrition, School of Nutritional Sciences and Dietetics, Tehran University of Medical Sciences (TUMS), Tehran, Iran; 2 Students’ Scientific Research Center (SSRC), Tehran University of Medical Sciences, Tehran, Iran; 3 Department of Clinical Biochemistry, School of Medicine, Tehran University of Medical Sciences, Tehran, Iran; 4 Department of Clinical Nutrition, School of Nutritional Sciences and Dietetics, Tehran University of Medical Sciences, Tehran, Iran; Tabriz University of Medical Sciences, ISLAMIC REPUBLIC OF IRAN

## Abstract

**Background:**

Previous studies showed the possible association between obesity, dietary pattern, and depressive symptoms. Due to the lack of enough data to confirm the association of obesity and depression in the Middle East, here, we aimed to explore the possible mediatory role of adipokines Galectin-3, transforming growth factor-beta (TGF-β), and endothelial plasminogen activator inhibitor (PAI-1) in the association between low carbohydrate diet (LCD) and depressive symptoms.

**Methods:**

A total of 256 women aged 17–56 years old were grouped based on their LCD score. Depression anxiety stress scales-21 (DASS-21) self-administered questionnaire was used to evaluate the three negative emotional states of stress, depressive symptoms, and anxiety. Body composition and dietary intake were assessed. Enzyme-linked immunosorbent assay (ELISA) was used to measure the serum levels of Galectin-3, TGF-β, and PAI-1.

**Results:**

No significant difference was observed regarding Galectin-3, TGF-β, and PAI-1 levels between the groups with dissimilar adherence to LCD or the groups with different levels of depressive symptoms (P>0.05). However, there was a negative association between LCD score as a covariant and depressive symptoms as an independent variable (P = 0.02) and remarkably, a regression model linear analysis using Galectin-3, TGF-β, and PAI-1 as confounding variables indicated the mediatory role of these adipokines in this association (P>0.05). In other words, adipokines eliminated the significance of the relationship between adherence to LCD and depressive symptoms.

**Conclusion:**

It seems that higher adherence to LCD is probably associated with a lower prevalence of depressive symptoms in obese adults through the mediatory role of adipokines.

## Introduction

Obesity is defined as an excessive accumulation of fat in white adipose tissue (WAT) with negative effects on health. It is a chronic disease resulting from a positive energy balance in conditions of energy excess or an imbalance between energy intake and expenditure [1–3]. Obesity is growing as a public health problem with a high prevalence in both developed and developing countries. It was estimated by World Health Organization (WHO) that almost a third of the adults are now classified as overweight (body mass index (BMI) > 25 kg/h2)) or obese (BMI ≥ 30) [4, 5].

A mental disorder clinically refers to the dysfunction of an individual’s cognition and emotional or behavioral control [6]. It is usually associated with significant distress in scotia, profession, work, and other important activities. Recent studies reported that psychiatric diseases such as major depressive disorder (MDD), dysthymia, manic, and hypomanic episodes are more prevalent in obese patients rather than in normal-weight people [7, 8].

Supporting evidence on obesity and associated comorbidities has led to understanding the role of adipose tissue as a highly active endocrine organ in controlling the related physiological and pathological processes [1]. Indeed, adipose tissue plays an important role in mediating the biological effects on metabolism and inflammation. Moreover, it maintains energy homeostasis and secretes various adipokines as powerful peripheral signals from adipose tissue to the brain in the long-term control of appetite and energy balance. Remarkably, adipokines are suggested as critical mediatory factors in the association between obesity and psychopathology [9, 10]. Since the macronutrient composition of diet including the balance of carbohydrates and proteins is a key factor in the regulation of overall body weight and metabolism, and it also regulates the secretion of adipokines, then, high carbohydrate and glycemic index in the diet may change the level of adipokines and affect their mediatory roles as reported elsewhere [11]. In this way, several studies indicated the positive effects of low carbohydrate diet (LCD) on short and long term weight loss programs, body composition, cardiovascular, and metabolic features i.e. abdominal fat, high-density lipoprotein cholesterol (HDL-C), fasting blood sugar (FBS), insulin, and blood pressure and also psychological outcomes in contrast to the diet with macronutrient composition [12–14]. Moreover, individuals with a higher LCD score have lost weight faster than ones using other conventional diets like low-fat diet or Mediterranean diet. Hence, LCD seems to be a more effective way to reach a quick treatment of obesity [15, 16].

Previously, a possible association between LCD and depressive symptoms mediated by adipokines has been investigated in obese people with mood disorders [17]. In particular, the role of adipokines Galectin-3, transforming growth factor-beta (TGF-β), and endothelial plasminogen activator inhibitor (PAI-1) in the pathophysiology of mental disorders such as depression has been reported before [18–20]. Having said that, although several studies examined the levels of these adipokines in depressed people, the mediatory role of them is still in its infancy. Remarkably, as far as we have known, studies on the association of LCD with Galectin-3, TGF-β, and PAI-1 have been studied less than other adipokines.

Hence, in the present study, we intended to investigate the relationship between adherence to LCD and depressive symptoms as well as assess the mediatory role of these three biological substances (Galectin-3, TGF-β, and PAI-1) among overweight and obese women. It should be considered that the reason for selecting only the female gender as our target group was in accordance with the previous studies reporting a sexually dimorphic pattern of circulating adipokines levels [21–23]. Moreover, despite growing attention to women’s health, there is still a pressing need to investigate this group of society to improve their health [10]. We should note that the present study may unveil some new avenues about obesity and depression as one of the most important physical and psychological reasons for disability in the world with respect to the role of these less studied adipokines.

## Material and methods

### Study population

We performed a cross-sectional study on 256 randomly selected healthy women aged 17–56 year old who referred to health centers in Tehran. Participants had a BMI with a range of 25–40 kg/m^2^. A self-administered questionnaire was provided from all participants for their health status and the exclusion criteria of the study were followed as a history of hypertension, cardiovascular disease, diabetes, thyroid disease, malignancy, mental disorder, any acute or chronic inflammatory disease, impaired renal and liver function, regular use of medications such as birth control pills, intake of alcohol or drug abuse, smoking, pregnancy and lactation period. Furthermore, we excluded those participants with chronic diseases affecting their diet as well as those who have followed an arbitrary special dietary regimen or with any bodyweight fluctuations over the past year. The study protocol was approved by the ethics committee of Tehran University of Medical Sciences (IR.TUMS.VCR.REC.1395.1234). All participants signed written informed consent. We have also obtained written informed consent from parents or guardians of the minors included in the study.

Blood samples were collected following overnight fasting and serum was centrifuged, liquated, and stored at a temperature of −80°C until the analysis was performed. All samples were analyzed using a single assay according to the manufacturer’s protocol. All measurements were taken at the Endocrinology & Metabolism Research Institute (EMRI) Bionanotechnology laboratory of Tehran University of Medical Science. Triglyceride (TG) and FBS were measured by Randox Laboratories Kit (Hitachi 902). The serum levels of Galectin-3 (Human Galectin-1, ELISA kit Crystal Company), TGF-β (Human TGF-BETA 1 Quantikine ELISA kit R&D System-USA), and PAI-1 (Human PAI-1 ELISA kit Crystal Company) were measured in triplicate.

### Anthropometry measurements (body composition analysis)

An impedance fat analyzer (InBody 720, Korea) was used to acquire weight, BMI, body fat mass (BFM), fat mass (FM), fat-free mass (FFM), fat percentage (%), and visceral fat area (VFA) of the subjects following a standardized procedure. In detail, a low-level electrical current was sent through the body into the electrodes of hands and feet. Then, the impedance of currents was measured to evaluate the body composition.

### Dietary assessment

Dietary intake and nutritional status over the past year were evaluated using a semi-quantitative food frequency questionnaire (FFQ). This procedure was designed according to the Willett study including a list of 147 food items along with a standard serving size for each nutrient [24]. The reliability and validity of this FFQ have been approved in 2010 in Iran [25]. FFQ data were analyzed by Nutritionist-4 software.

### Calculation of the LCD score

Data were collected from completed FFQ forms by participants in Tehran Health center. These data were used to calculate the LCD score based on the percent of energy intake. In this way, a higher LCD score means a lower carbohydrate intake and a higher fat and protein intake, demonstrating higher adherence to a low-carbohydrate-dietary pattern. On the other hand, a lower LCD score reflects a higher carbohydrate intake and a lower fat and protein intake, indicating lower adherence to a low-carbohydrate-dietary pattern [12].

Accordingly, we separated the study participants into eleven strata (based on their score from 0 to 10) for each component; i.e. carbohydrate, vegetable protein, refined grain, monounsaturated fatty acid (MUFA), and n3/n6 polyunsaturated fatty acid (PUFA) as a percentage of energy intake as well as fiber and glycemic load (GL). Women with the highest intake of vegetables, protein, MUFA, PUFA, and fiber in their macronutrient received 10 points and women with the lowest received 0 points. In contrast, those with the lowest level of refined grain, GL, and carbohydrate intake got 10 points and those with the highest intake of these macronutrients got 0 points. Dietary GL was estimated as total glycemic index × total available carbohydrate (g) per 100. We considered the percentage of consumed energy instead of absolute intake to decrease the bias resulting from under-reporting of food and representing the food components. For estimating the LCD score, we summed the total scores of these 7 components in which 0 showed the highest carbohydrate and the lowest protein and fat intake and 70 showed the lowest carbohydrate and the highest protein and fat intake [26]. Therefore, the highest score belonged to those who followed the pattern of LCD.

### Depressive symptoms assessment

The depression anxiety stress scales-21 (DASS-21) is a 42-item self-report questionnaire to evaluate the three negative emotional states of stress, depressive symptoms, and anxiety. To determine the total score for each subscale, a respondent indicates a 4-point scale with the degree of each item applied over the last week and the z-score can be used for values comparison. A normal state is defined as a z-score of 0.5, a mild state is a z-score of 0.5 to 1, a moderate state is a z-score of 1 to 2, a severe state is a z-score of 3, and an extremely severe state is often defined as a z-score more than 3 [27].

### IPAQ assessment

The International Physical Activity Questionnaire (IPAQ) is designed to provide data of walking and moderate or vigorous physical activity for a comparable evaluation, especially for research purposes. This questionnaire can be used by young and middle-aged adults. Here, we recorded the needed information about the physical activity of our study population according to the standard methods for adapting these data to our country’s study centers. For weekly physical activity assessment, a short form (9 items) was applied using a metabolic equivalent (MET) score for each type of activity. According to the protocol of IPAQ scoring, the MET score was demonstrated as 3 and 6 METs for moderate activities and as 6 METs for vigorous activities to show the overall physical activity. For reporting the total physical activity from all activity categories, MET scores from all these sub-components were summed and MET-minutes per week (MET-min/week) were estimated [28].

### Statistical analysis

Continuous variables with normal distribution were provided with mean ± standard error of mean (SEM) and skewed distributed variables were provided with median (IQR). ANOVA with Scheffe post hoc was used for normal distributed data, and Kruskal wails and Bonferroni correction post hoc were performed for non-normal distributed data. Then, ANCOVA analysis was used to remove the effect of potential confounders. The linear regression model analysis was used for finding the modulatory role of Galectin-3, TGF-β, and PIA levels on LCD score and depressive symptoms relationship. The stepwise method was also used for finding the modulatory role of Galectin-3, TGF-β, and PIA in the linear regression model. Multiple stepwise linear regression was performed to identify the best set predictors for these adipokines with different anthropometric and laboratory variables. Moreover, multinomial logistic regression was performed to ascertain the risk of depressive symptoms regarding the adipokines levels. P value < 0.05 was considered statistically significant. Statistical analysis was performed using SPSS version 22.0 (SPSS, Chicago, IL, USA).

## Results

### Study population characteristics

The anthropometric and laboratory characteristics of the study population are provided in Table 1.

**Table 1:** Anthropometric and laboratory characteristics of the study population.

Parameters	Minimum	Maximum	Mean	Std. Deviation
**Age (years)**	17	56	36	8.4
**Weight (kg)**	59.5	136.6	80.9	12.5
**Height (cm)**	142	179	161	5.9
**BMI (kg/m** ^**2**^ **)**	24.2	49.6	31	4.3
**Fat percentage (%)**	15	54.3	41.5	5.5
**Obesity degree percentage (%)**	29.4	231	143.7	21.5
**Waist circumference (cm)**	80.1	136	99	10
**Waist-hip ratio (cm)**	0.81	92	1.2	5.2
**Visceral fat (%)**	7	208.4	16.6	13.9
**BFM (kg)**	19.4	74.2	34	8.7
**FFM (kg)**	35.3	67.7	46.8	5.6
**FMI (kg/m** ^**2**^ **)**	6.9	26.9	13.2	3.4
**FBS (mg/dl)**	67	137	87.5	9.6
**TC (mg/dl)**	104	344	185.3	35.8
**TG (mg/dl)**	370	512	122.1	69.3
**hs-CRP (mg/L)**	0	22.7	4.3	4.6
**Energy (kcal)**	1029	4192.7	2613	751.7
**LCD score**	6	58	35	10.2
**TGF-β (pg/mL)**	32.9	494.7	78.6	48.6
**PAI-1 (pg/mL)**	0.5	202	16.1	29.9
**Galectin-3 (pg/mL)**	0.15	32.3	4.1	7.3

BMI, body mass index; BFM, body fat mass; FFM, fat-free mass; FMI, fat mass index; FBS, fasting blood sugar; TC, total cholesterol; TG, triglyceride; hs-CRP, high-sensitivity C-reactive protein; LCD, low carbohydrate diet; TGF-β, transforming growth factor-beta; PAI-1, plasminogen activator inhibitor-1.

We assessed the study variables among groups with different adherence to LCD by ANOVA test. First, the participants were grouped based on the LCD score as group 1 (n = 85), group 2 (n = 85), and group 3 (n = 84) in which group 3 showed the most adherence to LCD score compared to group 1. The results showed that there were statistically significant differences between the groups regarding weight (0.03), height (P = 0.02), FFM (P < 0.01), and depressive symptoms (P = 0.02). Hence, it was observed that participants with higher adherence to LCD had lower levels of depressive symptoms. However, there was no significant difference in terms of age, BMI, fat percentage, obesity degree percentage, waist circumference, waist-hip ratio, visceral fat, BFM, FMI, FBS, total cholesterol (TC), TG, HDL-C, high-sensitivity C-reactive Protein (hs-CRP), Galectin-3, TGF-β, and PAI-1 (P>0.05) (Table 2).

**Table 2:** Assessment of the study variables among groups with different adherence to LCD.

Variable	T1 (N = 85)	T2 (N = 85)	T3 (N = 84)	P value
**Age (years)**	36 ± 8	35 ± 8	38 ± 9	0.11
**Weight (kg)**	83.6 ± 3.4	81.4 ± 12.8	78.8 ± 10.1	0.03
**Height (cm)**	162 ± 6	162 ± 6	160 ± 6	0.02
**BMI (kg/m** ^**2**^ **)**	31.6 ± 4.9	31 ± 4	31.6 ± 3.9	0.23
**Fat percentage (%)**	41.3 ± 5.5	41.6 ± 5.5	41.6 ± 5.6	0.88
**Obesity degree percentage (%)**	146.8 ± 22.6	143.1 ± 2	142.2 ± 18.5	0.16
**Waist circumference (cm)**	98.6 ± 10.3	98.9 ± 9	99.3 ± 10.7	0.88
**Waist-hip ratio (cm)**	0.9 ± 0.04	0.9 ± 0.05	0.9 ± 0.05	0.53
**Visceral fat (%)**	15.9 ± 3.4	15.5 ± 3.4	15. 2 ± 3.2	0.35
**BFM (kg)**	35.4 ± 9.7	34 ± 8.6	32.7 ± 7.4	0.08
**FFM (kg)**	47.4 ± 5.5	47.8 ± 56.1	45.4 ± 5	<0.01
**FMI (kg/m** ^**2**^ **)**	12.7 ± 3	13.1 ± 2.7	12.9 ±3.2	0.66
**FBS (mg/dl)**	87.2 ±11.1	87.4 ± 8.5	888.1 ± 9.6	0.80
**TC (mg/dl)**	183.8 ± 37.5	183.1 ± 33.5	187.8 ± 38.3	0.66
**TG (mg/dl)**	127.8 ± 75.2	116.5 ±71.1	122.8 ± 65.6	0.59
**hs-CRP (mg/L)**	4.6 ±4.7	4.1 ±4.5	4 ±4.5	0.59
**Depressive symptoms**	5.8 ±4.9	5.9 ±4.5	4.2 ± 4.5	0.02
**TGF-β (pg/mL)**	75.6 ± 33.7	74.5 ± 34	83.6 ± 66.3	0.53
**PAI-1 (pg/mL)**	17.3 ± 26.1	15.5 ± 32.5	15.1 ± 30.3	0.91
**Galectin-3 (pg/mL)**	4.9 ± 7.8	2.1 ± 2.2	4.1 ± 7.8	0.42

In order to examine the association of LCD score with study variables and depressive symptoms, the participants were grouped based on their LCD score, group 1 (n = 85), group 2 (n = 85), and group 3 (n = 84). The three column in table is arranged by the adherence to LCD and the third group (T3) has a higher adherence compared to others (T3>T2>T1). LCD, low carbohydrate diet; BMI, body mass index; BFM, body fat mass; FFM, fat-free mass; FMI, fat mass index; FBS, fasting blood sugar; TC, total cholesterol; TG, triglyceride; hs-CRP, high-sensitivity C-reactive protein; TGF-β, transforming growth factor-beta; PAI-1, plasminogen activator inhibitor-1. A P value < 0.05 was considered as significant.

We diagnosed 46.45% of our participants had depressive symptoms by DASS-21 self-administered questionnaire. Accordingly, 157 subjects with a z-score of 0.5 were defined as normal, 93 subjects with a z-score of 0.5 to 2 were defined as patients with mild and moderate depressive symptoms, and 50 subjects with a z-score more than 2 were defined as patients with severe and extremely severe depressive symptoms based on the total score for each subscale of the DASS-21 self-administered questionnaire. Then, we analyzed the study variables among groups with different depressive symptoms, including normal participants, patients with mild and moderate depressive symptoms, and patients with severe and extremely severe depressive symptoms by ANOVA test. The results showed that there were statistically significant differences between the groups regarding TG (0.01), FMI (P = 0.05), and obesity degree percentage (P = 0.02), and waist circumference (P = 0.03). Moreover, there was no significant change due to the three states of depressive symptoms in age, weight, height, BMI, fat percentage, waist-hip ratio, visceral fat, BFM, FFM, FBS, TC, hs-CRP, Galectin-3, TGF-β, and PAI-1 (P>0.05) (Table 3).

**Table 3:** Assessment of the study variables among groups with different depressive symptoms.

Parameters	Normal (N = 157)	Mild and moderate depressive symptoms (N = 93)	Severe and extremely severe depressive symptoms (N = 50)	P value
**Age (years)**	36 ± 8	37 ± 8	37 ± 10	0.9
**Weight (kg)**	82 ± 13.2	79 ± 11.7	83.1 ± 12.4	0.14
**Height (cm)**	161 ± 6	161 ± 6	161 ± 6	0.96
**BMI (kg/m** ^**2**^ **)**	31.3 ± 4.5	30.3 ± 3.8^c^	32 ± 4.7^b^	0.08
**Fat percentage (%)**	41.8 ± 6.1	40.7 ± 4.8	42.4 ±5.1	0.18
**Obesity degree percentage (%)**	145.5 ± 21b	138.8 ± 22.4ac	148.8 ± 22.1b	0.02
**Waist circumference (cm)**	96.5 ± 8.4	102.6 ±12.4	98.9 ±9.9	0.03
**Waist-hip ratio (cm)**	0.9 ±0.05	0.9 ± 0.05	1.4 ± 6.3	0.84
**Visceral fat (%)**	17 ± 16.3	16.5 ± 14.8	16.2 ± 3.1	0.92
**BFM (kg)**	34.7 ± 9.2	32.5 ± 7.9	35.6 ± 8.7	0.08
**FFM (kg)**	46.9 ± 6	46.6 ± 5.4	47.3 ± 5.3	0.79
**FMI (kg/m** ^**2**^ **)**	13.3 ± 3.5	12.5 ± 3^c^	14 ± 3.7^b^	0.05
**FBS (mg/dl)**	87.4 ± 8.8	87.4 ± 9.8	87.7 ± 11.8	0.98
**TC (mg/dl)**	187.2 ± 35.5	182.8 ± 39.7	184.5 ± 29.5	0.69
**TG (mg/dl)**	119 ± 65.5	108.9 ± 55	149.2 ± 96.5	0.01
**hs-CRP (mg/L)**	3.7 ± 4.5	5.5 ± 4.4	4.2 ± 4.8	0.35
**TGF-β (pg/mL)**	50.8 ± 14.5	55.5 ± 16.4	101.2 ± 50.5	0.79
**PAI-1 (pg/mL)**	13.4 ± 31.5	12.5 ± 22.2	18.3 ± 31.5	0.6
**Galectin-3 (pg/mL)**	2.8 ± 6.6	3.3 ± 5.3	4.6 ± 7.9	0.69

47.66% of the participants had depressive symptoms. We divided them into two groups: (mild and moderate) and (severe and extremely severe). We analyzed the association of the study groups with all variables and LCD score by ANOVA test. BMI, body mass index; BFM, body fat mass; FFM, fat-free mass; FMI, fat mass index; FBS, fasting blood sugar; TC, total cholesterol; TG, triglyceride; hs-CRP, high-sensitivity C-reactive protein; TGF-β, transforming growth factor-beta; PAI-1, plasminogen activator inhibitor-1. A P value < 0.05 was considered as significant.

### Association between LCD score and depressive symptoms

We performed ANOVA test to define the relationship between depressive symptoms and LCD score. We used LCD score as a covariant and depressive symptom as an independent variable which revealed a significant association (P = 0.02) (confidence interval = 5.5 to 9.4) (beta ±SE = -0.1±0.03). After adjustment for age, BMI, energy, and IPAQ, this relationship remained significant (P = 0.02) (confidence interval = -0.1 to -0.08) (beta ±SE = -0.1±0.03) (Table 4).

**Table 4:** Association between depressive symptoms and LCD score.

	LCDS					
	Crude model			Adjusted model*		
	β ± SE	95%CI	P value	β ± SE	95%CI	P value
**Depressive symptoms**	−0.1±0.03	5.5–9.4	0.02	−0.1±0.03	−0.1 - −0.08	0.02

ANOVA test was used to assess the relationship between depressive symptoms and LCD score.

*Adjusted for age, BMI, energy, and IPAQ.

LCD, low carbohydrate diet; BMI, body mass index; IPAQ, international physical activity questionnaire. A P value < 0.05 was considered as significant.

### Mediatory role of Galectin-3, TGF-β, and PAI-1 in the relationship between LCD and depressive symptoms

Using the correlation method, by including Galectin-3, TGF-β, and PAI-1 as confounding variables along with the other confounders in the final model (which indicated a significant association between LCD and depressive symptoms) we observed that these three adipokines eliminated the significance of the relationship between adherence to LCD and depressive symptoms (P = 0.7, P = 0.4, and P = 0.4, respectively). Therefore, Galectin-3, TGF-β, and PAI-1 may be considered as mediatory adipokines. In other words, our results demonstrated that LCD may improve depressive symptoms through the mediatory effects of Galectin-3, TGF-β, and PAI-1 (Table 5).

**Table 5:** Mediatory effect of adipokines on the association between depressive symptoms and LCD score.

	Beta ±SE	T value	P value
**Galectin-3 (pg/mL)**	0.04±0.06	0.4	0.69
**TGF-β (pg/mL)**	-0.7±0.04	-0.8	0.4
**PAI-1 (pg/mL)**	-0.07±0.04	-0.9	0.39

LCD, low carbohydrate diet; TGF-β, transforming growth factor-beta; PAI-1, plasminogen activator inhibitor. A P value < 0.05 was considered as significant.

## Discussion

Proteins secreted by adipose tissue termed adipokines have several effects on many physiological processes such as controlling the food intake, energy homeostasis, insulin sensitivity, regulation of blood pressure, and even sending signals from adipose tissue to the brain. In the context of mood states, adipokines contribute to body weight dynamics [29]. Visceral fat releases several adipokines, including Galectin-3, TGF-β, and PAI-1 [30]. Since the association of obesity and adipokines with metabolic disturbances were reported before, and also there is a notable relationship between obesity, dietary pattern, and depressive symptoms [9–11], here, we investigated the possible role of Galectin-3, TGF-β, and PAI-1 in mediating the association between adherence to LCD and depressive symptoms among overweight and obese women. It should be noted that any change in adipokines levels may be a conceivable prospective regulation of the obesity-depression association [17].

Remarkably, in the present study, the regression model linear analysis revealed that Galectin-3, TGF-β, and PAI-1 may have some mediatory roles in the association between adherence to LCD and depressive symptoms among overweight and obese women. However, this conceivable association remains unknown and has not been well-defined yet. There are some plausible explanations regarding these results based on previous studies. For instance, in addition to the role of adipokines in metabolic regulation, they also work as a bidirectional communicator between the adipose system and hypothalamic pituitary adrenal (HPA) axis. Remarkably, it was reported that the most usual biological change in obesity and depression is dysregulation of the HPA axis [31]. Moreover, adipokines levels are reduced by the stimulatory effects of chronic or high-intensity stress and also are affected by glucocorticoids like cortisol [32]. In this manner, Taylor and MacQueen studied the role of adipokines as anti-obesity hormones and observed that cortisol is a possible pathophysiological mediator in the excess weight gain of their study population [33]. Evidence showed that weight gain and cortisol levels have a mutual influence on each other and obesity is followed by an early increase in the intracellular levels of cortisol in adipose tissue [34, 35]. Interestingly, it should be noted that adipokines may be dysregulated in mental disorders along with higher levels of cortisol and visceral obesity. For instance, the association of obesity and depression through regulating adipokines has been investigated in a recent study indicating their positive correlation, particularly in women. Additionally, a higher activity of the HPA axis was reported too which was associated with the secretion of adipokines. In addition, adipokines in this study regulated the HPA axis in some way, therefore, all of them were likely to affect the circulating levels of cortisol too [36]. Thus, according to previous studies, adipokines may regulate cortisol levels in depressed patients and be related to obesity risk factors, including high BMI and insulin resistance [37, 38].

Remarkably, previous studies showed the role of Galectin-3, TGF-β, and PAI-1 in the pathophysiology of depression [18–20]. As an instance, a recent study reported that depression in diabetes was linked to higher levels of Galectin-3 [39]. Another study demonstrated that MDD patients with high abdominal fat had higher levels of PAI-1. Indeed, PAI-1 might act as a crucial link between obesity and MDD [20]. Notably, to the best of the authors’ knowledge, no data are available on the association of LCD with depressive symptoms regarding Galectin-3, TGF-β, and PAI-1 levels. Hence, the important finding of the current study is that participants with higher adherence to LCD had lower levels of depressive symptoms through the mediatory effects of Galectin-3, TGF-β, and PAI-1. This cross-sectional study strengthened this concept that LCD may alter the possibility of depressive symptoms in obese people through the mediatory effects of these adipokines.

It should be noted that mood and weight loss due to diet are related to each other with an improvement in the mood after losing weight [40]. Food containing high proteins will produce more sense of fullness and less tiredness compared to food containing high carbohydrates. Moreover, dietary management like low calorie and fiber consumption may be effective in the regulation of adipokines levels too. Remarkably, mood and mental states can be affected by high-fat diets possibly through the variations of corticosterone levels [41–44].

Today, LCD is one of the remarkable diets which mainly focuses on proteins and fats. In this diet, due to the restriction of carbohydrates, fat oxidation is started to produce ketone bodies (KBs) which are fuel for the brain [45, 46] and can be used medically to control epilepsy and seizures [47]. Moreover, LCD has long-term effects on cognitive function in contrast to diets with enough carbohydrates [48, 49]. Previous similar studies showed the effective role of LCD in losing weight [11, 50]. Interestingly, a clinical study represented by Brinkworth G.D. *et al*. introduced LCD as an effective alternative dietary pattern for weight loss. Furthermore, they showed the effects of LCD compared to other restricted calorie diets on mood improvement. Therefore, LCD may be used as a mood stabilizer in depressive disorders [51]. However, the mechanism for mood improvement due to LCD is still unknown [52]. Remarkably, an elevated tendency for consuming delicious and high-calorie foods is a consequence of increased glucocorticoid levels due to chronic stress. Hence, the outcomes are an accumulation of visceral fat and a change in the levels of adipokines [53, 54]. In this context, it was reported that a ketogenic diet caused higher levels of TGF-β in spontaneously hypertensive rats [55]. It was also indicated that adherence to LCD improved the circadian rhythm by decreasing the level of Galectin-3 as a mediatory factor in this relationship [10]. Furthermore, individuals following LCD had lower PAI-1 levels along with other types of adipokines [56]. Here, it seems likely that Galectin-3, TGF-β, and PAI-1 produced by adipose tissue are affected by dietary pattern and may also have potential energy-mood mediatory effects. Although, their exact mechanism is poorly recognized.

Altogether, the present study emphasizes the importance of controlling obesity through LCD affecting both obesity and depressive symptoms. Here, for the first time we demonstrated that Galectin-3, TGF-β, and PAI-1 may act as a critical link between obesity and depressive symptoms, and as a consequence, they may represent a possible novel insight in the disease monitoring and treatment. However, the relatively small number of subjects in the sample size and also investigating subjects with the same-gender (256 females) are some limitations of the present study which deserve to be mentioned.

## Conclusion

In the current cross-sectional study, we found the mediatory role of circulating Galectin-3, TGF-β, and PAI-1 adipokines in the relationship between adherence to LCD and depressive symptoms among overweight and obese women. Indeed, it seems likely that LCD is associated with depressive symptoms by mediating the levels of these adipokines. Altogether, these adipokines may represent a potential link for obesity and depressive symptoms. However, further studies and clinical trials should be performed comprising the cellular and molecular states to clarify the exact mechanism of Galectin-3, TGF-β, and PAI-1 and also their effects on the association of obesity with mood disorders through following LCD.

## Supporting information

S1 Dataset (XLSX)
